# Resistant schizophrenia in a patient with oculocutaneous albinism successfully treated with clozapine

**DOI:** 10.1192/j.eurpsy.2022.1192

**Published:** 2022-09-01

**Authors:** A. Jelti, F. N’Sabi, F. Kennab, M. Barrimi

**Affiliations:** Mohammed VI University Hospital, Faculty of Medicine and Pharmacy, University Mohammed Premier, Department Of Psychiatry, Oujda, Morocco

**Keywords:** Oculocutaneous albinism, resistant schizophrenia, clozapine

## Abstract

**Introduction:**

Several studies have shown an association between oculocutaneous albinism and several neuropsychiatric entities, including schizophrenia.

We present through this work the first case of resistant schizophrenia described in this type of patient.

**Objectives:**

Demonstrate the safety of clozapine in albinos patients and discuss the linkage between the two disorders.

**Methods:**

We describe the case of a 23-year-old patient with oculocutaneous albinism who developed treatment-resistant schizophrenia, successfully treated with clozapine.

**Results:**

We received the patient referred by his private psychiatrist, after the absence of improvement despite the use of several first and second generation antipsychotics, alone and in combination, at therapeutic doses, with good compliance and for a duration of more than 3 months.

The onset of psychotic symptoms was at the age of 18 years, and the evolution was continuous.

On admission, the patient was agitated and assessment of thought content were difficult due to intellectual disorganization. The initial PANSS score was assessed at 120. The somatic examination was normal, except for generalized hypopigmentation and horizontal and rotatory nystagmus. A biological workup, MRI, and electroencephalogram revealed no abnormalities.

The diagnosis of resistant schizophrenia was retained and the patient was put on clozapine 12.5 mg/d with progressive titration to a dose of 400 mg at the 6th week, with marked improvement; the patient became motor calm, with understandable speech and adapted responses, the PANSS score at the 2nd month decreased markedly by 33 (from 120 to 87).

**Conclusions:**

This case report suggests that clozapine can be safely introduced in patients with oculocutaneous albinism.

**Disclosure:**

No significant relationships.

